# Current Ethanol Production Requirements for the Yeast *Saccharomyces cerevisiae*

**DOI:** 10.1155/2022/7878830

**Published:** 2022-08-13

**Authors:** Flávia da Silva Fernandes, Érica Simplício de Souza, Lívia Melo Carneiro, João Paulo Alves Silva, João Vicente Braga de Souza, Jacqueline da Silva Batista

**Affiliations:** ^1^Postgraduate Program in Genetics, Conservation and Evolutionary Biology-GCBEv, National Institute for Amazonian Research (INPA), Manaus, Brazil; ^2^Higher School of Technology, Amazonas State University (UEA), Manaus, Brazil; ^3^Department of Chemical Engineering, Engineering College of Lorena, University of São Paulo, Sao Paulo, Brazil; ^4^Mycology Laboratory, National Institute for Amazonian Research (INPA), Manaus, Brazil; ^5^Thematic Laboratory of Molecular Biology, National Institute for Amazonian Research (INPA), Manaus, Brazil

## Abstract

An increase in global energy demand has caused oil prices to reach record levels in recent times. High oil prices together with concerns over CO_2_ emissions have resulted in renewed interest in renewable energy. Nowadays, ethanol is the principal renewable biofuel. However, the industrial need for increased productivity, wider substrate range utilization, and the production of novel compounds leads to renewed interest in further extending the use of current industrial strains by exploiting the immense, and still unknown, potential of natural yeast strains. This review seeks to answer the following questions: (a) which characteristics should *S. cerevisiae* have for the current production of first- and second-generation ethanol? (b) Why are alcohol-tolerance and thermo-tolerance characteristics required? (c) Which genes are related to these characteristics? (d) What are the advances that can be achieved with the isolation of new organisms from the environment?

## 1. Introduction

The yeast *Saccharomyces cerevisiae* is undeniably the best studied and one of the most widely used eukaryotes in a wide variety of industrial processes such as ethanol production [1]. Currently, the annual production of alcohol worldwide is over 100 billion liters, with *S. cerevisiae* being the predominantly used industrial microorganism for ethanol production [2]. The yeast *S. cerevisiae* is the organism of choice for the industrial production of ethanol and, as such, represents the largest industrial biotechnological utilization of yeast.

The yeast *S. cerevisiae* has many desirable industrial properties such as rapid growth, efficient glucose anaerobic metabolism, high ethanol productivity, great yield, and high tolerance to different environmental stress factors, such as high ethanol concentration, low pH, and low oxygen level [3]. The use of existing or adapted industrial yeast strains in biotechnological and industrial fermentations is intensive; however, there is still much room for improvement since current industrial processes rarely exploit new natural strains [4].

The improvement in the production of first-generation ethanol is a process that involves the selection of yeasts with high fermentation speeds and dominance, long-lasting lifespans during the harvest, good fermentation capacity, elevated sugar-to-ethanol conversion rates, low output of glycerol, low foam levels, tolerance to high concentrations of substrate and ethanol, resistance to acidity and high temperatures, genetic stability, flocculence, good fermentation efficiency, high productivity, elevated cell growth speeds, elevated ethanol output, and substrate consumption speeds [5].

Improving second generation production includes isolating or developing microorganisms that ferment, in addition to glucose, pentose sugars that are abundant in lignocellulose hydrolysates, xylose, and L-arabinose, as well as microorganisms that can ferment different hydrolyzed sugars simultaneously and microorganisms that are resistant to inhibitors [3] and stressful conditions such as increased ethanol concentration and temperature [6]. The yeast *S. cerevisiae* has been widely studied and engineered for lignocellulosic valorization for second generation ethanol production [7] and high-value chemicals [8].

Despite of the efficient adaptation of the various *S. cerevisiae* strains used in these processes, there is still a great potential for either optimizing existing strains or exploiting the immense natural reservoir of environmental isolates [1]. However, there are a number of challenges common to yeasts during sugar fermentation due to increased temperature and ethanol levels. *S. cerevisiae* has limited tolerance to ethanol, and the maximum concentration that allows growth is 10% (p : v). Although *S. cerevisiae* yeasts are mesophilic (growth from 25°C to 30°C), often the temperatures in the distilleries reach 38°C [9]. Performing fermentation at higher temperatures using thermotolerant yeast could not only achieve a higher ethanol production with faster polysaccharide hydrolysis rates and shorter SSF (simultaneous saccharification and fermentation) times but could also reduce the cost of cooling and the rate of contamination [10].

This review provides an overview of studies with industrial and natural strains of *S. cerevisiae* for ethanol production and discusses the characteristics *S. cerevisiae* should have for current ethanol production, what advances can be achieved from the isolation of new organisms from the environment, why the characteristics of alcohol-tolerance and thermal tolerance are required, and which genes are related to these characteristics.

### 1.1. The Yeast *Saccharomyces cerevisiae*

**Figure 1:**
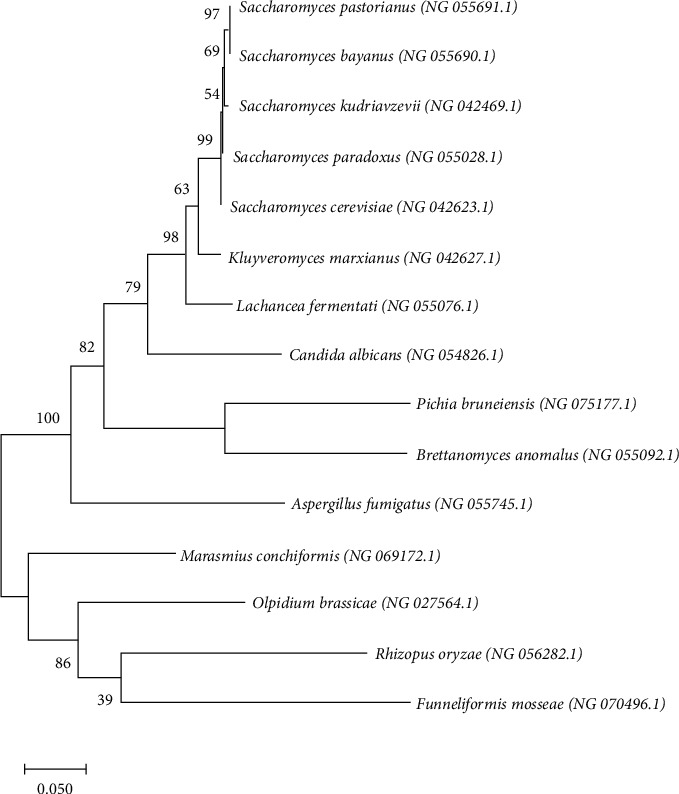
Phylogenetic tree constructed using the 28s rDNA sequences. Sequences are labeled with their database accession numbers. Support values are from Bayesian inference, and maximum likelihood analyses (values above and below the branches, respectively) were conducted in MEGA X software (molecular evolutionary genetics analysis: http://www.megasoftware.net/).

The yeast *S. cerevisiae* belongs to the group of Ascomycete yeasts (phylum: Ascomycota; subphylum: Saccharomycotina; class: Saccharomycetes; order: Saccharomycetales) [11]. One of the first publications on yeast taxonomy was made by Guilliermond [12], in which the genus *Saccharomyces* had 46 species in 6 highlighted groups according to their fermentative potential with sugars. Since that time, *Saccharomyces* has undergone important changes, especially the group *Saccharomyces sensu stricto*, which, in 1970, resulted in 41 species within the genus. According to Vaughan-Martin and Martini [13], the species included in the genus *Saccharomyces* are *S*. *arboricolus*, *S*. *bayanus* (*S*. *bayanus var.bayanus* and *S. bayanus var.uvarum*), *S. cariocanus*, *S. cerevisiae*, *S. kudriavzevii*, *S. mikatae*, *S. paradoxus*, and *S. pastorianus* (Figure 1).

Archaeological evidence exists regarding the production of a fermented beverage in China in 7000 BC and of wine in Iran and Egypt in 6000 BC and 3000 BC, respectively [14–16]. Since that time, these fermentation technologies have expanded from Mesopotamia to the rest of world. It is assumed that, at the beginning, fermentation was driven by the natural occurrence of yeasts in the substrate/environment, with probable exchange and interaction of yeasts between different fermentation processes. It is not known when the practice of conscious use of yeasts began in the manufacture of beverages. However, it was only at the end of the 19^th^ century that this habit was gradually replaced by selected cultures containing single or combined strains [17, 18].

In this regard, Martini [19] concluded that wine yeast comes mainly from wineries since isolation of the strain from nature or plants is rare [20] and concluded that this species is domesticated. As for its application in technological processes, scientific knowledge in the area has advanced since the first microscopic observation of yeasts by Antonie van Leeuwenhoek in 1680 and the studies by Louis Pasteur in 1858, who conclusively proved the primary catalytic role of yeasts in wine fermentation [21, 22].

*Saccharomyces* species are the most important commercial yeasts and have been studied as models of a eukaryotic organism for many years [23]. A typical *S. cerevisiae* haploid cell has genomic DNA of approximately 12,000 kb, divided into 16 linear chromosomes with a size ranging from approximately 200 to 2200 kb [24, 25]. *S. cerevisiae* also presents important characteristics for laboratory work, such as nonpathogenicity, easy growth, and is susceptible to transformation techniques and isolation of mutants, among others. It is the first eukaryotic organism with a sequenced genome in the *Saccharomyces* Genome Project [26], which is a project that monitors the presence of more than 6,608 ORFs (open reading frames or open reading matrix) which 5,797 encoded polypeptides. Before that, more than a third of these ORFs had no known function, even four years after their discovery. *S. cerevisiae* has 4,666 proteins with functions annotated in the *Saccharomyces* Genome Database [27].

The importance of the genus *Saccharomyces* in the technological development of fermentation processes and as a model in scientific studies is unquestionable; however, little is known about its natural history, ecology, genomic processes, and evolution, which are essential factors for understanding the biology of these microorganisms. The evolution of the yeasts of the genus *Saccharomyces* shows a direct relationship of each species with the natural environment since populations that coexist in the same habitat develop phenotypic convergence, while competition between species and lineages from different niches is rare or unstable. These interactions define metabolic traits and survival strategies, and a determining factor is the different use and availability of resources in each environment [22, 28]. Phylogenetic analyses point to events in evolutionary development that mark the adaptation and favoring of certain species to growth at higher or lower temperatures. In the study by Lip et al. [29], a phenotypic screening of 12 industrial yeast strains and the laboratory strain CEN.PK113-7D was performed at cultivation temperatures between 12°C and 40°C which revealed significant differences in maximum growth rates and temperature tolerance. The authors observed differences in biomass and ethanol yields in glucose, biomass protein and storage carbohydrates, and biomass yields in ATP between strains and culture temperatures. The increase in temperature tolerance coincided with the greater energy efficiency of cell growth, indicating that temperature intolerance is a result of energy-wasting processes, such as increased turnover of cellular components due to temperature-induced damage.

The biological characteristics of *S. cerevisiae* have been reviewed by Landry et al. [30], and this work also included genetic characteristics. In short, *S. cerevisiae* is a diplontic yeast with a high degree of clonal reproduction. It is also homothallic, which confers the possibility of regenerating a diploid cell from a haploid, and this can be interpreted as a form of genome renewal. This mechanism may be responsible for the high rate (28%) of homozygote strains found in vineyards [31].

**Figure 2:**
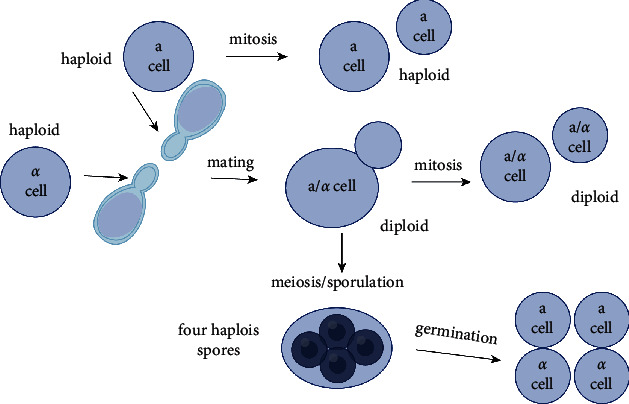
The life cycle of budding yeasts. ^*∗*^Image created using Biorender.

The life cycle of budding yeasts goes through asexual and sexual reproductive cycles. The budding yeast reproduces both as haplontic (haploid) and diplontic (diploid) cells during the asexual life cycle through mitosis (Figure 2). Haploid cells of opposite mating types (*a* or *α*) can go on to mate (conjugate) and reform diploid cells [32]. However, under highly stressful conditions, such as nutrient starvation, haploid cells will die, while diploid cells undergo meiosis to form haploid spores through sporulation [33, 34].

The budding yeast grows and divides through an asymmetric budding process. During mitosis, the daughter cell begins to form as a small bud on the tip of the mother cell. In metaphase, one set of sister chromatids moves into the bud. The continued growth of the bud eventually becomes a separated daughter cell. Budding yeasts have all the typical eukaryotic cell cycle stages of *G*1, *S*, *G*2, and *M* (mitosis) phases, which can be recognized by DNA content, nuclear morphology, and bud morphology. The yeast cells are found in fluctuating environments in the wild and are often subjected to shortages of food. *S. cerevisiae* cells are therefore likely to spend much of their time in a nondividing state known as quiescence [35], in which conditions become favorable and the yeast is able to grow on a modest array of fermentable and nonfermentable carbon sources (mostly six-carbon sugars). The availability of nutrients is likely to result in a rapid, mitotic clonal expansion of diploid yeast cells.

### 1.2. First- and Second-Generation Ethanol Production

Nowadays, bioethanol is the main source of renewable biofuel with about 27 billion gallons produced globally in 2021 [36], most of which is obtained from corn starch and sugarcane. The United States is the world's leading producer with about 15 billion gallons. When combined with the 7.5 billion gallons produced from sugar cane by Brazil, the two countries produce about 82% of the world's ethanol. Brazil is the second largest producer and consumer of ethanol in the world [36].

**Figure 3:**
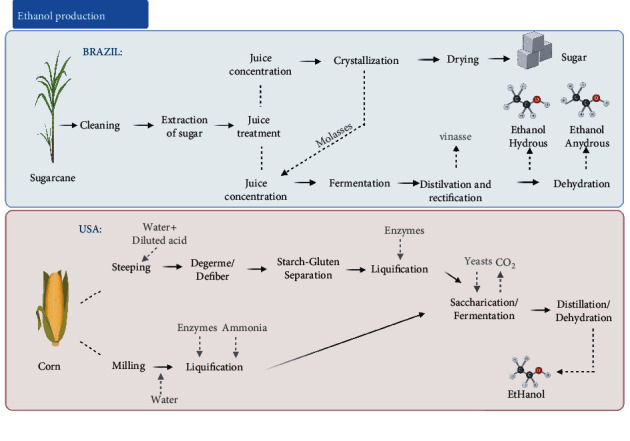
Ethanol production process using corn (in USA) and sugarcane (in Brazil) as substrate. ^*∗*^Image was created using Biorender.

The Melle-Boinot fermentation process is the most popular in Brazil [37] (Figure 3). It is based on yeast recovery from fermented wine by centrifugation, allowing the reuse of yeast after treatment to avoid bacterial contamination. The produced wine proceeds to distillation columns, in which ethanol is separated from the wine based on the different boiling points of the components in this mixture. The wine is decomposed into two streams: phlegm (vapors with 40–50°GL) and vinasse (liquid stream with less than 0.03°GL used as fertilizer in the crop fields). Phlegm follows the rectification process to achieve 96°GL and results in hydrous ethanol. Ethanol in hydrated form can be used as a final product, e.g., vehicular fuel, or proceed to the dehydration process. The anhydrous ethanol (99.7°GL) is most commonly obtained by using cyclohexane for dehydration, and addition of cyclohexane results in a ternary mixture with water and ethanol, whose boiling point is lower than the initial binary mixture. After separation, the dehydrated product is recovered and reused. Anhydrous ethanol is widely used in the chemical industry as a raw material for the manufacture of esters, ethers, solvents, paints, varnishes, cosmetics, or it can be mixed with gasoline as an additive for this vehicular fuel.

In 2005, the United States surpassed Brazil and became the world's number one ethanol producer. Dry milling is dominant in the country and accounts for almost 90% of total USA ethanol production [38]. In the dry milling process (Figure 3), the whole corn kernel is ground into a powder and mixed with water to form a mash, to which liquefying enzymes (amylase) are added to break down the starch into simple sugars. Ammonia is also added for pH control and as a nutrient for the yeast in the subsequent fermentation step. The mash is then boiled to avoid bacterial contamination and then proceeds to the saccharification step, in which liquefied starch is hydrolyzed to glucose with saccharifying enzymes (glucoamylase). After cooling, the mash proceeds to the subsequent steps of ethanol production. The glucose-rich mash obtained after saccharification step advances to the fermentation process, which normally takes about 30–40 h at mesophilic temperatures [39, 40]. In order to reduce the residence time of reactors, the application of simultaneous saccharification and fermentation (SSF) is widely used, in which glucoamylase and yeast (*Saccharomyces cerevisiae*) are added simultaneously [41]. The resulting mixture, containing about 15% ethanol and solids from the grain and added yeast, is pumped to a multicolumn distillation system, where the ethanol is separated from the remaining stillage. From distillation, 93–95% pure ethanol is obtained, which is dehydrated to approximately 99% pure ethanol in a molecular sieve system [39].

For the American process, the industrial strain ethanol Red [42] is the most widely employed yeast. In Brazil, two important strains of the species *S. cerevisiae* designated CAT-1 and PE-2 are used. More recently, the yeasts PE-2, CAT-1, BG-1, and SA-1 are being used in more than 70% of all Brazilian distilleries [43].

### 1.3. Yeast Characteristics Needed for First-Generation Ethanol Production

In the production of first-generation ethanol from corn, the starch must be solubilized and then subjected to two enzymatic steps to obtain fermentable sugars [44, 45]. The use of *α*-amylase in starch-based industries has been prevalent for many decades, and a number of microbial sources exist for the efficient production of this enzyme; however, only a few selected strains of fungi and bacteria meet the criteria for commercial production [46]. In order to obtain a new strain of yeast that can produce ethanol directly from starch without the need for a separate saccharification process that supports the stressors during fermentation, studies on methods that improve the fermentation potential of existing strains or isolate new strains with important characteristics are increasingly necessary.

The American process imposes a high concentration of ethanol, while the Brazilian process imposes acid treatment, cell recycling, high temperatures, competition with indigenous yeasts and bacteria, and also osmotic stress due to the high concentrations of sugar at the beginning [6]. In this process, a good industrial strain must be sufficiently robust to respond well to environmental variations in this environment, without altering its fermentative characteristics [3, 6].

The characteristics required by a yeast used in the production of first-generation ethanol are those of fast growth, efficient anaerobic glucose metabolism, high ethanol productivity, high yield, and high tolerance to several environmental stress factors such as high ethanol levels, lower pH, and low oxygen. The isolation of new environmental strains of *S. cerevisae* with characteristics such ethanol tolerance, thermotolerance, among other characteristics, are necessary in order to increase the yield in the ethanol industry [47]. This is why exploring the existing natural diversity of strains in the search for yeasts with traits that can contribute to a phenotype with tolerance to specific processes during production is essential.

### 1.4. Second-Generation Ethanol Production and Yeast Requirements

**Figure 4:**
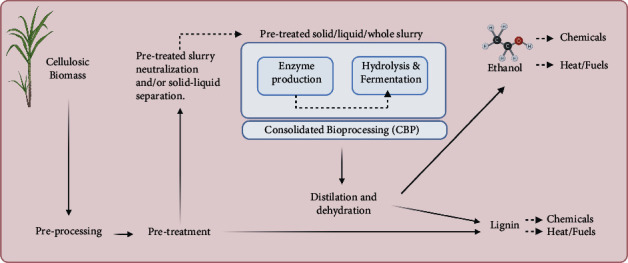
Flowchart showing the production of second-generation ethanol. ^*∗*^Image was created using Biorender.

Based on the International Energy Agency (IEA) definition, the term second-generation biofuel refers to biofuels produced from lignocellulosic biomass, i.e., from cellulose-hemicellulose-lignin composed feedstock [48] (Figure 4). The utilization of lignocellulosic biomass for second-generation ethanol (2GE) production is preferable over sugar and starch-based first-generation ethanol (1GE) production because of the absence of competition with food production [49, 50]. Examples of lignocellulose include agricultural wastes (corn stover, wheat, or rice straw), sugarcane bagasse, grass, domestic waste, and dedicated energy crops (Chinese silver grass and switchgrass) [51]. 2GE is an attractive technology that increases the production of fuels per hectare [52].

The consolidated bioprocessing (CBP) of lignocellulosic biomass is sustainable strategy what connects the three steps of lignocellulosic bioethanol production, namely, enzyme production, enzymatic saccharification, and sugar fermentation, followed by biological conversion of the pentoses and hexoses to valuable products using a single organism or a consortium [53, 54]. Remarkable efforts to engineer *S. cerevisiae* for that purpose are noted in several studies such as then in the study by Davison et al. [55], in which high yields of corn cob ethanol were achieved by *S. cerevisiae* YI13 coexpressing EGII (*Trichoderma reesei* endoglucanase) and BGLI (*Saccharomycopsis fibuligera* beta-glucosidase). In another study, a strategy was developed by constructing a cell-surface displayed consortium using two engineered yeasts (Y5/XynII-XylA (codisplaying two types of xylanases) and Y5/EG-CBH-BGL (codisplaying three types of cellulases)) that heterologously expressed functional lignocellulolytic enzymes to convert pretreated corn stover to ethanol [56].

Several governments and private entities have financed new plants for the production of second-generation ethanol, which may be integrated or not in the first-generation ethanol production process or renovated existing plants in preparation for this new technology in order to optimize productivity. In the US, the most important companies are DuPont Cellulosic ethanol LLC and Poet-DSM Advanced Biofuels LLC-Libertya Project, both using corn cobs for ethanol production. These companies produce 113.6 and 75 million liters of cellulosic ethanol per year, respectively. In Canada, the most productive company is Enerkem Alberta Biofuels LP, which produces 38 million liters of cellulosic ethanol per year from separated domestic solid waste [57].

The world's first large-scale ethanol plant was built in Guangxi (China) by COFCO in 2007 [58]. Italy, anticipating world demand and using state-of-the-art technology, is now home to the largest cellulosic ethanol plant in the world. An initiative of the company Beta Renewables, which is a global leader in second-generation biofuels and part of the Mossi and Ghisolfi group. The plant has a structure that is capable of producing 75 million liters of cellulosic ethanol per year, using wheat straw, rice straw, and a kind of giant sugarcane (*Arundo donax*) as raw materials [59].

In some parts of Europe, especially France and Italy, grapes have become a raw material for fuel ethanol production since ethanol can be made from surplus wine. The Norwegian company Borregaard Industries AS-ChemCell ethanol has the capacity to produce 20 million liters of cellulosic ethanol per year from wood pulp residues. Nigeria and Ghana are also establishing cassava plantations for ethanol production. In Brazil, two plants are already producing second-generation ethanol: the companies Granbio and Raízen. Granbio, in the state of Alagoas and Raízen, in the state of São Paulo. Together, they have a production capacity of 100 million liters per year. As such, from 2023, Brazil intends to increase, in a summarized manner, the synthesis of second-generation ethanol in order to reach production of 2.5 billion liters per year [59].

The process of converting lignocellulosic biomass into fermentable sugars to produce second-generation ethanol (2GE) involves four sequential steps: (1) pretreatment, to breaks down the plant cell wall, by disrupting the cellulose from lining and hemicellulose, and exposes it to the enzymes [60]; (2) hydrolysis, to degrade cellulose fibres and hemicellulose into sugar monomers [61], (3) fermentation, to convert sugars into ethanol, and (4) distillation, a stage in which the solution obtained from the fermentation process is then distilled to separate high quality ethanol from the aqueous solution [62] (Figure 4). However, these processes can be performed independently (SHF: separate hydrolysis and fermentation) or combined (SSF: simultaneous saccharification and fermentation) [63].

One of the factors to have an efficient bioethanol production process is the maximum reduction of the formation of inhibitor compounds during pretreatment. These substances are weak acids (acetic, formic, and levulinic acids), furan derivatives (furfural and 5-hydroxymethylfurfural (5-HMF)), and phenolic compounds (such as syringic acid, vanillin, ferulic acid, vanillic, and coumaric acid) [64]. Some techniques on detoxifying the hydrolysates by removing the toxic chemical residues have been reported, including physical (evaporation and membrane separation) and chemical (overliming with calcium hydroxide, activated charcoal treatment, ion exchange resins, neutralization, and organic solvent extraction) [65–67]. Other strategies include changes in fermentation methodologies and metabolic engineering [68]. These inhibitory compounds are cytotoxic and inhibit microbial growth, metabolism, and ethanol yield [69]. Some studies report that the concentrations of furfural found in fermentations of hydrolysates of bagasse, rice husk, and *Bactris gasipaes* can vary between 0.10–0.36, 0.05–0.17, and 0.009–0.02, respectively. These authors also reported that HMF concentrations found in fermentations of hydrolysates of bagasse, rice husk and *Bactris gasipaes* fermentations ranged from 0.03–0.07, 0.10–0.21, and 0.06–0.18, respectively. The variations in the concentrations of Furfural and HMF in these studies were mainly due to the hydrolysis time and type of substrate used [70, 71].

There is still much scope for developing superior industrial yeast strains that could address the challenges and limitations of cellulosic ethanol production. However, the limited pool of available industrial strains represents only a small share of the actual genetic diversity present in nature. At the same time, numerous recent studies have highlighted the enormous unexploited diversity of *Saccharomyces* stricto sensu yeast [71, 72] and that many natural strains exhibit superior complex traits, such as inhibitor and temperature tolerance, that can be beneficial to the industry. It would thus serve academia and the industry at large to devote equal efforts towards improving existing industrial strains for second-generation ethanol production, but simultaneously explore the vast diversity available in nature as well. More extensive genotyping and phenotyping of native strains will support identifying strains and species with novel and/or improved industrially driven properties. The more extensive use of molecular techniques to study and enhance complex traits such as cofermentation of hexoses and pentoses, inhibitor tolerance, osmotolerance, and thermotolerance are crucial [4, 73].

Some common challenges of yeasts can be overcome by using ethanol-tolerant and thermotolerant yeast. Ethanol fermentation at high temperature is a beneficial process as it selects thermo-tolerant microorganisms and does not require the expenditure involved with cooling costs or with the cellulase enzyme [4]. The thermotolerance of the ethanol red strain of the species *S. cerevisiae* was analyzed in the study to Pinheiro et al. [72], and the strain was subjected to high temperatures. Under these conditions, the strain increased the expression of proteins involved in sterol and glycogen synthesis, together with Hsp104p, known to play a role crucial in adapting to heat. In another study, Techaparin et al. [74] analyzed that the highly expressed genes encoding heat shock proteins, HSP82 and SSA4, potentially play an important role in helping *S. cerevisiae* KKU-VN8 deal with various stresses that occur during fermentation of high temperature, leading to greater efficiency in ethanol production.

Another important factor is that, in contrast to corn or sugar cane, cellulosic biomass is more difficult to convert into fermentable sugars than corn or sugar cane because it has five-carbon sugars, mainly xylose, due to the presence of lignin, a highly recalcitrant network polymer of aromatic alcohols that account for 17–25% of common cellulosic biomass [75] and because cellulose is much more resistant to hydrolysis than starches and simple oligosaccharides. The first obstacle can be overcome through the selection and/or engineering of microorganisms capable of carrying out alcoholic fermentation of xylose and other pentoses. The traditional ethanol fermenters, *S. cerevisiae* and *Z. mobilis* cannot utilize pentoses but can only ferment glucose to ethanol. *Pichia stipitis*, *Candida shehatae*, and *Pachysolen tannophilus* are the major pentose e fermenting yeasts that have been used extensively [73]. On the other hand, there are microorganisms that produce lignocellulolytic enzymes, with *Trichoderma reesei* and *Aspergillus Niger* being the most important industrial producers [76]. Other microorganisms are being studied in the last years such as *Myceliophtora thermophila* (cellulase and xylanase), *Aspergillus ibericus* (cellulase, *β-*glucosidase, and xylanase), *Coriolus versicolor* (Mn peroxidase, lignin peroxidase, and laccases) [77–79].

All forms of microorganisms have undergone experimental modifications resulting in what are called genetically modified organisms (GMOs). GMOs have been developed to improve the resistance of microorganisms to inhibitors generated during pretreatment, as well as their tolerance to ethanol and high concentrations of sugar and to increase the range of sugars (hexoses and pentoses) consumed, making the ethanol process more efficient [69, 80]. The genetic modifications have been widely done in three microorganisms, such as *S. cerevisiae* (yeast), *Z. mobilis* (bacterium), and *E. coli* (bacterium) [81]. Some examples of GMO *Saccharomyces cerevisiae* yeast strains for bioethanol production are ER-Xpress, FT 858L, CelluX (Leaf Technologies), rich yeast + GA (Richmond Chemicals), Innova Drive (Novozymes), and Xyloferm (Lallemand/Taurus Energy AB). The use of GM strains of *S. cerevisiae* is absolutely necessary for optimizing the conversion of both hexose and pentose sugars to ethanol. There is great promise for synthetic biology in such processes, and more generally, for yeast biotechnology in the future [2].

The significant amount of knowledge about *S. cerevisiae* in databases makes this yeast an attractive platform for genetic improvement and metabolic engineering [82]. In the study by Cadete et al. [83] was observed that *S. cerevisiae* TMB 3504, which expresses XYL1.2p from *Sp. passalidarum*, showed significant ethanol yield and productivity (0.40 vs. 0.34 g g − 1 CDW). In another example, Kobayashi et al. [84] observed an overexpression of all enzymes involved in nonoxidative PPP (via pentose phosphate), including RKI1, RPE1, TKL1, and TAL1, and improved xylose uptake rates and ethanol yields in recombinant *S. cerevisiae* expressing the pathway. And, in another study, an overexpression of XYL2 in the *S. cerevisiae* SF7-Ft3 strain consistently led to better utilization of xylose by various enzymatic hydrolates of lignocellulose residues and increased bioethanol yields (% dry matter) and concentrations (g/L) at 11%–42% [85].

Research on bioethanol production has several axes, which include the discovery of new natural microorganisms (or the “construction” of genetically modified ones) that produce ethanol in significant concentrations of the final product and high volumetric productivities and/or small amounts antagonistic to the metabolites of the ethanol (i.e., glycerol). One of the problems in yeast fermentation of bioethanol is the ability to ferment pentose sugars. *S. cerevisiae* is the most commonly used in bioethanol production; however, it can only ferment hexoses, though not pentoses [86]. From this perspective, several works have focused on pentose consuming organisms (e.g., xylose and arabinose), which are sugars that are found in significant quantities in lignocellulosic biomass [25, 35–37].

### 1.5. What Are the Genes and Metabolic Pathways Related to the Characteristics Required in Yeast for Ethanol Production?

During the industrial production of first- and second-generation ethanol, yeasts are submitted to several stressing factors such as high ethanol concentration and high temperatures. Temperature has long been known to affect the metabolism of yeasts, and fermentation at high temperature becomes more prone to bacterial contamination. In addition, the yeast is more sensitive to alcohol toxicity, leading to the formation of metabolites such as trehalose, glycerol, acetic acid, and succinic acid, among others [6, 87–89].

The decrease in yeast cell viability at higher temperatures is also due to the accumulation of intracellular ethanol, which produces cell toxicity and alters the membrane structure, thus decreasing its functionality [88, 90–92]. According to Dorta [9], the yeast *S. cerevisiae* has limited tolerance to the ethanol, whose maximum concentration that allows growth is 10% (p : v), and high concentrations of ethanol can affect the structure of the enzymes, resulting in decreased catalytic activity [93]. Therefore, determining factors, such as high ethanol concentration and high temperature, must be improved to increase the productive capacity of yeast strains during industrial production of first- and second-generation ethanol.

*S. cerevisiae* is known to employ many stress-responsive pathways in order to adapt to drastic changes in the environment [94]. Data reported by Teixeira et al. [95] indicate that the expression of the FPS1 (farnesyl diphosphate synthase 1) gene contributes to the reduction of alcohol accumulation within the cell during the fermentation process, suggesting that FPS1 may have a role in regulating the level of intracellular ethanol and that the increased expression of this gene can increase the yeast's ability to produce high concentrations of alcohol.

Another protein, called ASR1 (alcohol sensitive RING/PHD finger1protein) is encoded by the ASR1/YPR093C gene. Under alcoholic stress, this protein modifies its intracellular distribution in the cytoplasm and accumulates in the nucleus, transmitting an alcoholic stress signal from the plasma membrane to the nucleus. Thus, it becomes a key element in ethanol tolerance and is essential for the normal development of the cell in a medium containing high concentrations of alcohol [94, 96].

Other essential genes identified as determinants of yeast resistance to inhibitory concentrations of ethanol are identified in the following list [95]:

- BDP1 (essential subunit of RNA polymerase III transcription factor (TFIIIB)
- CSL4 (subunit of the exosome, which is an essential complex present in both the nucleus and cytoplasm that mediates RNA processing and degradation)
- CWC25 (component of a complex containing Cef1p, involved in pre-mRNA splicing)
- HTS1 (cytoplasmic and mitochondrial histidine tRNA synthetase)
- IRR1 (subunit of the cohesin complex, which is required for sister chromatid cohesion during mitosis and meiosis and interacts with centromeres and chromosome arms)
- MED8 (subunit of the RNA polymerase II mediator complex associates with core polymerase subunits to form the RNA polymerase II holoenzyme)
- MPE1 (essential conserved subunit of CPF (cleavage and polyadenylation factor); plays a role in 3′ end formation of mRNA via the specific cleavage and polyadenylation of pre-mRNA; contains a putative RNA-binding zinc knuckle motif)
- PRP11 (subunit of the SF3a splicing factor complex, required for spliceosome assembly)
- RRP3 (involved in rRNA processing; required for maturation of the 35S primary transcript of pre-rRNA and for cleavage leading to mature 18S rRNA)
- SPP381 (mRNA splicing factor, component of U4/U6/U5 tri-snRNP)
- TFC1 (one of six subunits of the RNA polymerase III transcription initiation factor complex (TFIIIC); part of the TauA globular domain of TFIIIC that binds DNA at the BoxA promoter sites of tRNA and similar genes)
- FHL1 (putative transcriptional regulator with similarity to DNA-binding domain of *Drosophila* forkhead; required for rRNA processing)
- ARC35 (subunit of the ARP2/ARP3 complex, which is required for the motility and integrity of cortical actin patches)
- IDI1 (isopentenyl diphosphate:dimethylallyl diphosphate isomerase (IPP isomerase); catalyzes an essential activation step in the isoprenoid biosynthetic pathway
- NAT2 (N-*α*-acetyltransferase; transfers acetyl group from acetyl coenzyme A to the N-terminal methionine residues of proteins)
- SIS1 (type II HSP40 cochaperone that interacts with the HSP70 protein Ssa1p)
- STS1 (protein that interacts with the karyopherin Srp1p; may have a role with Srp1p in ubiquitin-mediated protein degradation)
- TOM40 (component of the TOM (translocase of outer membrane) complex, responsible for recognition and initial import steps for all mitochondrially directed proteins) [95]

Recent studies have been conducted to find thermotolerance-conferring pathways in *S. cerevisiae*, and these suggest the participation of several genes that are essential for achieving a high-temperature growth strain. Among these genes, RSP5 encoding ubiquitin ligase [97], TPS1, TPS2, and NTH1 that are involved in trehalose metabolism and ADH1 and CDC19 that are involved in the glycolytic pathway have been described in association with the increment of temperature tolerance [74].

The heat-shock response is a well-known molecular mechanism that makes cells more thermotolerant. In *S. cerevisiae*, the 3′ adenosine-dependent protein kinase and 5′-cyclic monophosphate c AMP (PKA) signaling pathway has been referred to as being a thermotolerance regulator. The cAMP/PKA pathway controls a variety of processes including the stress response [98]. The level of intracellular cAMP is regulated by adenylate cyclase (Cyr1p), which converts ATP to cAMP [99]. Depending on the CDC25p activity, monomeric G proteins (Ras1p and Ras2p) control Cyr1p activity. This is a membrane-bound guanine nucleotide exchange factor (GEF) that activates RAS1p and RAS2p by stimulating GDP release and GTP binding [100, 101]. The lowest level of cAMP initiates stress-responsive transcriptional activators such as Msn2p and Msn4p, resulting in stress tolerance [98].

Much still can be studied and genetically developed so that superior industrial yeast strains can face all the obstacles of producing cellulosic ethanol more efficiently. However, the limited set of available industrial strains represents only a small portion of the actual genetic diversity present in nature.

### 1.6. Environmental Isolation of *Saccharomyces cerevisiae*

The number of yeasts being discovered is increasing year on year. It is assumed that only 1% of yeast species are currently known, which represents approximately 1500 species. The total number of yeast species on Earth is estimated at 150,000 [102]. The diversity of yeast species in particular niches is determined by their ability to use different carbon sources and their nutritional selectivity for presenting great habitat specialization [103] and that many natural strains exhibit superior complex traits, such as inhibitor and temperature tolerance, that can be beneficial to the industry. Ethanol-tolerant and thermotolerant strains that can resist stresses can be isolated from natural resources such as soil, water, plants, and animals. This is because cells adapt to their environment over time via natural selection.

Recently, numerous studies have highlighted the enormous unexploited diversity of *Saccharomyces stricto sensu* yeast [104, 105], some studies have been carried out using environmental sources to isolate *S. cerevisiae* from fruits such as grape berries, mangoes, pineapples and orange peel, tree bark (*Quercus rubra, Tapirira guianensis* (*Tapirira*)), and fermented musts (Table1).

The environmental isolation of new yeast strains can lead to advances in the production of first- and second-generation ethanol. The production of first-generation ethanol requires yeast strains that not only produce ethanol directly from starch without the need for a separate saccharification process but also withstand stressors such as high ethanol levels and temperature during fermentation. The isolation of robust microbial strains that can grow and produce ethanol from at least glucose and xylose and that have tolerance of inhibitors and thermotolerance are crucial in the production process of second generation ethanol [4] and significantly influence the final yield in this process.

## 2. Conclusion

*S. cerevisiae* is the most used organism for 1st and 2nd generation ethanol production, but improvements are still needed, first generation ethanol production requires yeast strains that can produce ethanol directly from starch without the need for a saccharification process separated and that can withstand stressors such as high levels of ethanol and high temperatures during fermentation. Second-generation ethanol from lignocellulose will require the development of robust strains of *S. cerevisiae* that can grow and produce ethanol from at least glucose and xylose and that exhibit thermotolerance and tolerance to inhibitors such as phenolic compounds, furans, and weak acids.

Significant advances have already been achieved by combining beneficial traits from different lineages using adaptation and hybridization, as well as targeting specific traits through genetic engineering; however, the limited set of available industrial lineages represents only a small part of the current genetic diversity present in nature. Several recent studies have highlighted the enormous unexplored diversity of the yeast *Saccharomyces* stricto sensu, which has superior complex characteristics that could be beneficial to the alcohol industry.

Functional genomics is a powerful tool for directing metabolic changes to increase the rate and yield of ethanol production. Proteomic analysis of xylose fermentations has already revealed 22 proteins such as Adh2p, Ald4p, and Ald6p, showing significantly higher levels compared to glucose fermentation. Proteins such as ASR1 and FPS1 are essential for normal cell development in a medium containing high concentrations of alcohol and high ethanol production and that the highly expressed genes encoding heat shock proteins, HSP82, SSA4, and HSP104p, are known to play a crucial role in heat adaptation in *S. cerevisiae*.

However, overcoming major limitations such as incomplete substrate catabolism, low titers of heterologous protein expression, thermotolerance, ethanol tolerance, and impediment due to the accumulation of inhibitors/toxic byproducts is still a challenge. Science must cooperate both in improving existing industrial strains and in developing new phenotypes by exploiting the vast biodiversity available.

## Figures and Tables

**Table 1:** Different strategies used for the isolation of *Saccharomyces cerevisiae* from environmental sources.

Origin	Isolation technique	Isolated use	Reference
Grapes-spontaneously fermented musts	15 to 20 grapes were placed in 150 mL of MEM malt extract medium and cultivated for 10 days at 23°C	Search for isolates with new oenological properties for wine production	[106]

Spontaneously fermented musts	The must fermentation process was carried out in a 1000 L barrel, at 18°C	Search for supply of isolates with new oenological properties for wine production	[106]

Pineapple and orange peel	1 gram of sample was soaked in 250 ml of YMM yeast maintenance medium at 30°C for 3 days	High-potential, stress-tolerant, ethanol-producing yeasts	[107]

Cucumber jangajji	Three yeast strains were fortified from cucumber jangajji using a YM medium at 25°C for 48 h	Probiotics and important crops for affected foods	[108]

Bark of three tree species: *Quercus rubra*, unidentified tree. *Tapirira guianensis*	2 g of each sample was inoculated into flasks with RE medium and, after turbidity, aliquots were seeded in YMA with 8% ethanol	Second-generation ethanol industrial processes	[109]

Mango	Spontaneous mango fermentation took place for 7 days, and every 24 hours the sample was diluted, seeded on GPY agar medium and incubated at 30°C for 3 days	Cognac production	[110]

Palm wine	Beverage samples (1 mL) were directly diluted and plated in medium (YPD) and incubated at 30°C for 3–5 days	Evaluation of the genetic diversity and population structure of yeasts	[111]

Distilleries in northeast Brazil	Must samples were plated onto WLN medium containing nalidixic acid and ampicillin (both at 50 *μ*g ml^−1^) after appropriate dilutions	Ethanol production	[112]

Distillery wastes, sewage and algal bloom and dairy wastes	The samples were mixed with YEPD broth and incubated at 40°C at 150 rpm. After 24 h, 100 *μ*l of the diluted samples was spread on YEPD agar plates and incubated at 40°C for 48 h.	Ethanol production	[113]

## Data Availability

Information used for the elaboration of this review article originates from works published previously in the literature, and all the data are included within the article.

## References

[1] Parapouli M., Vasileiadis A., Afendra A.-S., Hatziloukas E. (2020). *Saccharomyces cerevisiae* and its industrial applications. *AIMS Microbiology*.

[2] Walker G. M., Walker R. S. K. (2018). Enhancing yeast alcoholic fermentations. *Advances in Applied Microbiology*.

[3] Dmytruk K. V., Kurylenko O. O., Ruchala J., Abbas C. A., Sibirny A. A. (2017). Genetic improvement of conventional and nonconventional yeasts for the production of first-and second-generation ethanol. *Biotechnology of Yeasts and Filamentous Fungi*.

[4] Favaro L., Jansen T., van Zyl W. H. (2019). Exploring industrial and natural *Saccharomyces cerevisiae* strains for the bio-based economy from biomass: the case of bioethanol. *Critical Reviews in Biotechnology*.

[5] de Vasconcelos J. N. (2015). Ethanol fermentation. *Sugarcane*.

[6] Basso L. C., De Amorim H. V., De Oliveira A. J., Lopes M. L. (2008). Yeast selection for fuel ethanol production in Brazil. *FEMS Yeast Research*.

[7] Cunha J. T., Soares P. O., Baptista S. L., Costa C. E., Domingues L. (2020). Engineered *Saccharomyces cerevisiae* for lignocellulosic valorization: a review and perspectives on bioethanol production. *Bioengineered*.

[8] Baptista S. L., Costa C. E., Cunha J. T., Soares P. O., Domingues L. (2021). Metabolic engineering of *Saccharomyces cerevisiae* for the production of top value chemicals from biorefinery carbohydrates. *Biotechnology Advances*.

[9] Dorta C., Oliva-Neto P., de-Abreu-Neto M. S., Nicolau-Junior N., Nagashima A. I. (2006). Synergism among lactic acid, sulfite, pH and ethanol in alcoholic fermentation of *Saccharomyces cerevisiae* (PE-2 and M-26). *World Journal of Microbiology and Biotechnology*.

[10] Wiegel J., Ljungdahl L. G., Demain A. L. (1985). The importance of thermophilic bacteria in biotechnology. *Critical Reviews in Biotechnology*.

[11] Suh S.-O., Blackwell M., Kurtzman C. P., Lachance M.-A. (2006). Phylogenetics of Saccharomycetales, the ascomycete yeasts. *Mycologia*.

[12] Guilliermond A. (1912). *Les Levures*.

[13] Vaughan-Martini A., Lachance M.-A., Kurtzman C. P., Zubkova K. (1971). *The Yeasts*.

[14] McGovern P. E., Zhang J., Tang J. (2004). Fermented beverages of pre-and proto-historic China. *Proceedings of the National Academy of Sciences*.

[15] McGovern P. E., Hartung U., Badler V. R., Glusker D. L., Exner L. J. (1997). The beginnings of winemaking and viniculture in the ancient near east and Egypt. *Expedition*.

[16] Cavalieri D., McGovern P. E., Hartl D. L., Mortimer R., Polsinelli M. (2003). Evidence for *S. cerevisiae* fermentation in ancient wine. *Journal of Molecular Evolution*.

[17] Sicard D., Legras J.-L. (2011). Bread, beer and wine: yeast domestication in the Saccharomyces sensu stricto complex. *Comptes Rendus Biologies*.

[18] Steensels J., Verstrepen K. J. (2014). Taming wild yeast: potential of conventional and nonconventional yeasts in industrial fermentations. *Annual Review of Microbiology*.

[19] Martini A. (1993). Origin and domestication of the wine yeast *Saccharomyces cerevisiae*. *Journal of Wine Research*.

[20] Sniegowski P. D., Dombrowski P. G., Fingerman E. (2002). *Saccharomyces cerevisiae* and Saccharomyces paradoxus coexist in a natural woodland site in North America and display different levels of reproductive isolation from European conspecifics. *FEMS Yeast Research*.

[21] Pretorius I. S. (2000). Tailoring wine yeast for the new millennium: novel approaches to the ancient art of winemaking. *Yeast*.

[22] Liti G., Carter D. M., Moses A. M. (2009). Population genomics of domestic and wild yeasts. *Nature*.

[23] Madigan M. T., Martinko J. M., Bender K. S., Buckley D. H., Stahl D. A. (2016). *Microbiologia de Brock-14^a^ Edição*.

[24] Bergman L. W. (2001). *Growth and Maintenance of Yeast. Two-Hybrid System*.

[25] Kellis M., Birren B. W., Lander E. S. (2004). Proof and evolutionary analysis of ancient genome duplication in the yeast *Saccharomyces cerevisiae*. *Nature*.

[26] Goffeau A., Barrell B. G., Bussey H. (1996). Life with 6000 genes. *Science*.

[27] Wiederhold E., Veenhoff L. M., Poolman B., Slotboom D. J. (2010). Proteomics of *Saccharomyces cerevisiae* organelles. *Molecular & Cellular Proteomics*.

[28] Spor A., Nidelet T., Simon J., Bourgais A., de Vienne D., Sicard D. (2009). Niche-driven evolution of metabolic and life-history strategies in natural and domesticated populations of *Saccharomyces cerevisiae*. *BMC Evolutionary Biology*.

[29] Lip K. Y. F., García-Ríos E., Costa C. E. (2020). Selection and subsequent physiological characterization of industrial *Saccharomyces cerevisiae* strains during continuous growth at sub-and-supra optimal temperatures. *Biotechnology Reports*.

[30] Landry C. R., Townsend J. P., Hartl D. L., Cavalieri D. (2006). Ecological and evolutionary genomics of *Saccharomyces cerevisiae*. *Molecular Ecology*.

[31] Otterstedt K., Larsson C., Bill R. M. (2004). Switching the mode of metabolism in the yeast *Saccharomyces cerevisiae*. *EMBO Reports*.

[32] Neiman A. M. (2005). Ascospore formation in the yeast *Saccharomyces cerevisiae*. *Microbiology and Molecular Biology Reviews*.

[33] Herskowitz I. (1988). Life cycle of the budding yeast *Saccharomyces cerevisiae*. *Microbiological Reviews*.

[34] Katz Ezov T., Chang S. L., Frenkel Z. (2010). Heterothallism in *Saccharomyces cerevisiae* isolates from nature: effect of HO locus on the mode of reproduction. *Molecular Ecology*.

[35] Gray J. V., Petsko G. A., Johnston G. C., Ringe D., Singer R. A., Werner-Washburne M. (2004). Sleeping beauty: quiescence in *Saccharomyces cerevisiae*. *Microbiology and Molecular Biology Reviews*.

[36] Renewable Fuel Association (RFA) (2022). *Annual World Fuel Ethanol Production*.

[37] Leal M. (2010). Technological evolution of sugarcane processing for ethanol and electric power generation. *Sugarcase Bioethanol R&D Product Sustain*.

[38] United States Department of Agriculture (USDA) (2016). *Natl Nutr Database Stand Ref Release 2016*.

[39] Lee S. (2007). Ethanol from corn. *Handbook of Alternative Fuel Technologies*.

[40] Nowak J., Szambelan K., Miettinen H., Nowak W., Czarnecki Z. (2008). Effect of the corn grain storage method on saccharification and ethanol fermentation field. *Acta Scientiarum Polonorum, Technologia Alimentaria*.

[41] Devantier R., Pedersen S., Olsson L. (2005). Characterization of very high gravity ethanol fermentation of corn mash. Effect of glucoamylase dosage, pre-saccharification and yeast strain. *Applied Microbiology and Biotechnology*.

[42] Kumar D., Singh V. (2016). Dry-grind processing using amylase corn and superior yeast to reduce the exogenous enzyme requirements in bioethanol production. *Biotechnology for Biofuels*.

[43] Parashar D., Satyanarayana T. (2017). Engineering a chimeric acid-stable *α*-amylase-glucoamylase (Amy-Glu) for one step starch saccharification. *International Journal of Biological Macromolecules*.

[44] de Moraes L. M. P., Astolfi Filho S., Ulhoa C. J. (1999). Purification and some properties of an *α*-amylase glucoamylase fusion protein from *Saccharomyces cerevisiae*. *World Journal of Microbiology and Biotechnology*.

[45] Toksoy Öner E. (2006). Optimization of ethanol production from starch by an amylolytic nuclear petite *Saccharomyces cerevisiae* strain. *Yeast*.

[46] Mobini-Dehkordi M., Afzal Javan F. (2012). Application of alpha-amylase in biotechnology. *Journal of Biology and Today’s World*.

[47] Mohd Azhar S. H., Abdulla R., Jambo S. A. (2017). Yeasts in sustainable bioethanol production: a review. *Biochemistry and Biophysics Reports*.

[48] Bacovsky D. (2010). How close are second generation biofuels?. *Biofuels, Bioproducts and Biorefining*.

[49] Sanchez O. J., Cardona C. A. (2008). Trends in biotechnological production of fuel ethanol from different feedstocks. *Bioresource Technology*.

[50] Nigam P. S., Singh A. (2011). Production of liquid biofuels from renewable resources. *Progress in Energy and Combustion Science*.

[51] Kumar M., Singhal A., Thakur I. S. (2016). Comparison of submerged and solid state pretreatment of sugarcane bagasse by Pandoraea sp. ISTKB: enzymatic and structural analysis. *Bioresource Technology*.

[52] Dias M. O. S., Junqueira T. L., Cavalett O. (2013). Biorefineries for the production of first and second generation ethanol and electricity from sugarcane. *Applied Energy*.

[53] Lynd L. R., Zyl W., McBride J. E., Laser M. (2005). Consolidated bioprocessing of cellulosic biomass: an update. *Current Opinion in Biotechnology*.

[54] Chandel A. K., Gonçalves B. C. M., Strap J. L., da Silva S. S. (2015). Biodelignification of lignocellulose substrates: an intrinsic and sustainable pretreatment strategy for clean energy production. *Critical Reviews in Biotechnology*.

[55] Davison S. A., Keller N. T., van Zyl W. H., den Haan R. (2019). Improved cellulase expression in diploid yeast strains enhanced consolidated bioprocessing of pretreated corn residues. *Enzyme and Microbial Technology*.

[56] Chen L., Du J.-L., Zhan Y.-J., Li J.-A., Zuo R.-R., Tian S. (2018). Consolidated bioprocessing for cellulosic ethanol conversion by cellulase–xylanase cell-surfaced yeast consortium. *Preparative Biochemistry & Biotechnology*.

[58] Hu R., Lin L., Liu T., Liu S. (2010). Dilute sulfuric acid hydrolysis of sugar maple wood extract at atmospheric pressure. *Bioresource Technology*.

[59] UNCTAD (2016). *Second Generation Biofuel Markets: State of Play, Trade and Developing Country Perspectives*.

[60] Rooni V., Raud M., Kikas T. (2017). Technical solutions used in different pretreatments of lignocellulosic biomass: a review. *Agronomy Research*.

[61] Menind A., Oper L., Hovi M., Kers J., Tutt M., Kikas T. (2012). Pretreatment and usage of pulp and paper industry residues for fuels production and their energetic potential. *Agronomy Research Biosystem Engineering*.

[62] Rocha-Meneses L., Raud M., Orupõld K., Kikas T. (2019). Potential of bioethanol production waste for methane recovery. *Energy*.

[63] Santos J. R. A., Lucena M. S., Gusmão N. B., Gouveia E. R. (2012). Optimization of ethanol production by *Saccharomyces cerevisiae* UFPEDA 1238 in simultaneous saccharification and fermentation of delignified sugarcane bagasse. *Industrial Crops and Products*.

[64] Pienkos P. T., Zhang M. (2009). Role of pretreatment and conditioning processes on toxicity of lignocellulosic biomass hydrolysates. *Cellulose*.

[65] Jurado M., Prieto A., Martínez-Alcalá Á., Martínez Á. T., Martínez M. J. (2009). Laccase detoxification of steam-exploded wheat straw for second generation bioethanol. *Bioresource Technology*.

[66] Ch A. K., Chan E. S., Rudravaram R., Narasu M. L., Rao L. V., Ravindra P. (2007). Economics and environmental impact of bioethanol production technologies: an appraisal. *Biotechnology and Molecular Biology Reviews*.

[67] Villarreal M. L. M., Prata A. M. R., Felipe M. G. A., Almeida Silva J. (2006). Detoxification procedures of eucalyptus hemicellulose hydrolysate for xylitol production by Candida guilliermondii. *Enzyme and Microbial Technology*.

[68] Larsson S., Cassland P., Jönsson L. J. (2001). Development of a *Saccharomyces cerevisiae* strain with enhanced resistance to phenolic fermentation inhibitors in lignocellulose hydrolysates by heterologous expression of laccase. *Applied and Environmental Microbiology*.

[69] Oreb M., Dietz H., Farwick A., Boles E. (2012). Novel strategies to improve co-fermentation of pentoses with D-glucose by recombinant yeast strains in lignocellulosic hydrolysates. *Bioengineered*.

[70] Martín C., Marcet M., Almazán O., Jönsson L. J. (2007). Adaptation of a recombinant xylose-utilizing *Saccharomyces cerevisiae* strain to a sugarcane bagasse hydrolysate with high content of fermentation inhibitors. *Bioresource Technology*.

[71] Fernandes F., Farias A., Carneiro L. (2021). Dilute acid hydrolysis of wastes of fruits from Amazon for ethanol production. *AIMS Bioengineering*.

[72] Pinheiro T., Lip K. Y. F., García-Ríos E. (2020). Differential proteomic analysis by SWATH-MS unravels the most dominant mechanisms underlying yeast adaptation to non-optimal temperatures under anaerobic conditions. *Scientific Reports*.

[73] Wirawan F., Cheng C.-L., Lo Y.-C. (2020). Continuous cellulosic bioethanol co-fermentation by immobilized Zymomonas mobilis and suspended Pichia stipitis in a two-stage process. *Applied Energy*.

[74] Techaparin A., Thanonkeo P., Klanrit P. (2017). High-temperature ethanol production using thermotolerant yeast newly isolated from greater Mekong subregion. *Brazilian Journal of Microbiology*.

[75] Van Maris A. J. A., Abbott D. A., Bellissimi E. (2006). Alcoholic fermentation of carbon sources in biomass hydrolysates by *Saccharomyces cerevisiae*: current status. *Antonie van Leeuwenhoek*.

[76] de Paula R. G., Antoniêto A. C. C., Nogueira K. M. V. (2019). Extracellular vesicles carry cellulases in the industrial fungus Trichoderma reesei. *Biotechnology for Biofuels*.

[77] Mishra V., Jana A. K. (2019). Sweet sorghum bagasse pretreatment by Coriolus versicolor in mesh tray bioreactor for selective delignification and improved saccharification. *Waste and Biomass Valorization*.

[78] Perez C. L., Casciatori F. P., Thoméo J. C. (2019). Strategies for scaling-up packed-bed bioreactors for solid-state fermentation: the case of cellulolytic enzymes production by a thermophilic fungus. *Chemical Engineering Journal*.

[79] Filipe D., Fernandes H., Castro C. (2020). Improved lignocellulolytic enzyme production and antioxidant extraction using solid state fermentation of olive pomace mixed with winery waste. *Biofuels, Bioproducts and Biorefining*.

[80] Kim J.-H., Block D. E., Mills D. A. (2010). Simultaneous consumption of pentose and hexose sugars: an optimal microbial phenotype for efficient fermentation of lignocellulosic biomass. *Applied Microbiology and Biotechnology*.

[81] Zabed H., Sahu J. N., Boyce A. N., Faruq G. (2016). Fuel ethanol production from lignocellulosic biomass: an overview on feedstocks and technological approaches. *Renewable and Sustainable Energy Reviews*.

[82] Nevoigt E. (2008). Progress in metabolic engineering of *Saccharomyces cerevisiae*. *Microbiology and Molecular Biology Reviews*.

[83] Cadete R. M., de Las Heras A. M., Sandström A. G. (2016). Exploring xylose metabolism in Spathaspora species: XYL1. 2 from Spathaspora passalidarum as the key for efficient anaerobic xylose fermentation in metabolic engineered *Saccharomyces cerevisiae*. *Biotechnology for Biofuels*.

[84] Kobayashi Y., Sahara T., Ohgiya S., Kamagata Y., Fujimori K. E. (2018). Systematic optimization of gene expression of pentose phosphate pathway enhances ethanol production from a glucose/xylose mixed medium in a recombinant *Saccharomyces cerevisiae*. *AMB Express*.

[85] He B., Hao B., Yu H. (2022). Double integrating XYL2 into engineered *Saccharomyces cerevisiae* strains for consistently enhanced bioethanol production by effective xylose and hexose co-consumption of steam-exploded lignocellulose in bioenergy crops. *Renewable Energy*.

[86] Kumar A., Singh L. K., Ghosh S. (2009). Bioconversion of lignocellulosic fraction of water-hyacinth (*Eichhornia crassipes*) hemicellulose acid hydrolysate to ethanol by Pichia stipitis. *Bioresource Technology*.

[87] Lafon-Lafourcade S., Carre E., Ribéreau-Gayon P. (1983). Occurrence of lactic acid bacteria during the different stages of vinification and conservation of wines. *Applied and Environmental Microbiology*.

[88] Hallsworth J. E. (1998). Ethanol-induced water stress in yeast. *Journal of Fermentation and Bioengineering*.

[89] Mattenberger F., Sabater Muñoz B., Hallsworth J. E., Fares M. A. (2017). Glycerol stress in S accharomyces cerevisiae: cellular responses and evolved adaptations. *Environmental Microbiology*.

[90] Ding J., Huang X., Zhang L., Zhao N., Yang D., Zhang K. (2009). Tolerance and stress response to ethanol in the yeast *Saccharomyces cerevisiae*. *Applied Microbiology and Biotechnology*.

[91] Stanley D., Bandara A., Fraser S., Chambers P. J., Stanley G. A. (2010). The ethanol stress response and ethanol tolerance of *Saccharomyces cerevisiae*. *Journal of Applied Microbiology*.

[92] Kitichantaropas Y., Boonchird C., Sugiyama M., Kaneko Y., Harashima S., Auesukaree C. (2016). Cellular mechanisms contributing to multiple stress tolerance in *Saccharomyces cerevisiae* strains with potential use in high-temperature ethanol fermentation. *AMB Express*.

[93] Bell A. N. W., Magill E., Hallsworth J. E., Timson D. J. (2013). Effects of alcohols and compatible solutes on the activity of *β*-galactosidase. *Applied Biochemistry and Biotechnology*.

[94] Ding J., Huang X., Zhao N., Gao F., Lu Q., Zhang K.-Q. (2010). Response of *Saccharomyces cerevisiae* to ethanol stress involves actions of protein Asr1p. *Journal of Microbiology and Biotechnology*.

[95] Teixeira M. C., Raposo L. R., Mira N. P., Lourenço A. B., Sá-Correia I. (2009). Genome-wide identification of S accharomyces cerevisiae genes required for maximal tolerance to ethanol. *Applied and Environmental Microbiology*.

[96] Betz C., Schlenstedt G., Bailer S. M. (2004). Asr1p, a novel yeast ring/PHD finger protein, signals alcohol stress to the nucleus. *Journal of Biological Chemistry*.

[97] Shahsavarani H., Sugiyama M., Kaneko Y., Chuenchit B., Harashima S. (2012). Superior thermotolerance of *Saccharomyces cerevisiae* for efficient bioethanol fermentation can be achieved by overexpression of RSP5 ubiquitin ligase. *Biotechnology Advances*.

[98] Görner W., Durchschlag E., Martinez-Pastor M. T. (1998). Nuclear localization of the C2H2 zinc finger protein Msn2p is regulated by stress and protein kinase a activity. *Genes & Development*.

[99] Matsumoto K., Uno I., Oshima Y., Ishikawa T. (1982). Isolation and characterization of yeast mutants deficient in adenylate cyclase and cAMP-dependent protein kinase. *Proceedings of the National Academy of Sciences*.

[100] Broek D., Samiy N., Fasano O. (1985). Differential activation of yeast adenylate cyclase by wild type and mutant RAS proteins. *Cell*.

[101] Broek D., Toda T., Michaeli T. (1987). The *S. cerevisiae* CDC25 gene product regulates the RAS/adenylate cyclase pathway. *Cell*.

[102] Barriga E. J. C., Libkind D., Briones A. I., Iranzo J., Portero P., Roberts I. (2011). Yeasts biodiversity and its significance: case studies in natural and human-related environments, ex situ preservation, applications and challenges. *Chang Divers Chang Environ*.

[103] Phaff H. J., Starmer W. T. (1987). 5 yeasts associated with plants, insects and yeasts. *Yeast Genetics*.

[104] Dujon B., Sherman D., Fischer G. (2004). Genome evolution in yeasts. *Nature*.

[105] Gallone B., Steensels J., Prahl T. (2016). Domestication and divergence of *Saccharomyces cerevisiae* beer yeasts. *Cell*.

[106] Šuranská H., Vránová D., Omelková J. (2016). Isolation, identification and characterization of regional indigenous *Saccharomyces cerevisiae* strains. *Brazilian Journal of Microbiology*.

[107] Nasir A., Rahman S. S., Hossain M. M., Choudhury N. (2017). Isolation of *Saccharomyces cerevisiae* from pineapple and orange and study of metal’s effectiveness on ethanol production. *European Journal of Microbiology and Immunology*.

[108] Lee N.-K., Hong J.-Y., Yi S.-H., Hong S.-P., Lee J.-E., Paik H.-D. (2019). Bioactive compounds of probiotic *Saccharomyces cerevisiae* strains isolated from cucumber jangajji. *Journal of Functional Foods*.

[109] Beato F. B., Bergdahl B., Rosa C. A., Forster J., Gombert A. K. (2016). Physiology of *Saccharomyces cerevisiae* strains isolated from Brazilian biomes: new insights into biodiversity and industrial applications. *FEMS Yeast Research*.

[110] Gobira R. M., Gobira P. S. S. C., Ferreira N. R., Monteiro R. C., Silva S. H. M. da, Souza A. B. (2017). Molecular identification of *Saccharomyces cerevisiae* strain isolated from spontaneous fermentation of mango (*Mangifera indica l.*). *Pulp*.

[111] Tra Bi C. Y., Amoikon T. L. S., Kouakou C. A. (2019). Genetic diversity and population structure of *Saccharomyces cerevisiae* strains isolated from traditional alcoholic beverages of Côte d’Ivoire. *International Journal of Food Microbiology*.

[112] Filho E. A. d S., de Melo H. F., Antunes D. F. (2005). Isolation by genetic and physiological characteristics of a fuel-ethanol fermentative *Saccharomyces cerevisiae* strain with potential for genetic manipulation. *Journal of Industrial Microbiology and Biotechnology*.

[113] Pandey A. K., Kumar M., Kumari S. (2019). Evaluation of divergent yeast genera for fermentation-associated stresses and identification of a robust sugarcane distillery waste isolate *Saccharomyces cerevisiae* NGY10 for lignocellulosic ethanol production in SHF and SSF. *Biotechnology for Biofuels*.

